# Deep Learning-Based Computed Tomography Perfusion Imaging to Evaluate the Effectiveness and Safety of Thrombolytic Therapy for Cerebral Infarct with Unknown Time of Onset

**DOI:** 10.1155/2022/9684584

**Published:** 2022-05-09

**Authors:** Minlei Hu, Ning Chen, Xuyou Zhou, Yanping Wu, Chao Ma

**Affiliations:** ^1^Department of Neurology, Affiliated Hospital of Jiaxing University, The First Hospital of Jiaxing, Jiaxing 314000, Zhejiang, China; ^2^Department of Radiology, Affiliated Hospital of Jiaxing University, The First Hospital of Jiaxing, Jiaxing 314000, Zhejiang, China; ^3^Department of Rehabilitation Medicine, Zhuji People's Hospital of Zhejiang Province, Zhuji 311800, Zhejiang, China

## Abstract

This study was aimed to discuss the effectiveness and safety of deep learning-based computed tomography perfusion (CTP) imaging in the thrombolytic therapy for acute cerebral infarct with unknown time of onset. A total of 100 patients with acute cerebral infarct with unknown time of onset were selected as the research objects. All patients received thrombolytic therapy. According to different image processing methods, they were divided into the algorithm group (artificial intelligence algorithm-based image processing group) and the control group (conventional method-based image processing group). After that, the evaluations of effectiveness and safety of thrombolytic therapy for the patients with acute cerebral infarct in the two groups were compared. The research results demonstrated that artificial intelligence algorithm-based CTP imaging showed significant diagnostic effects and the image quality in the algorithm group was remarkably higher than that in the control group (*P* < 0.05). Besides, the overall image quality of algorithm group was relatively higher. The differences in the National Institute of Health stroke scale (NIHSS) scores for the two groups indicated that the thrombolytic effect on the algorithm group was superior to that on the control group. Thrombolytic therapy for the algorithm group showed therapeutic effects on neurologic impairment. The symptomatic intracranial hemorrhage rate of the algorithm group within 24 hours was lower than the hemorrhage conversion rate of the control group, and the difference between the two groups was 14%. The data differences between the two groups showed statistical significance (*P* < 0.05). The results demonstrated that the safety of guided thrombolytic therapy for the algorithm group was higher than that in the control group. To sum up, deep learning-based CTP images showed the clinical application values in the diagnosis of cerebral infarct.

## 1. Introduction

Cerebral infarction with unknown time of onset, known as “ischemic stroke,” is a common disease of cerebral stroke, accounting for approximately 60%∼80% among cerebral diseases. It arises from long-term insufficient cerebral blood supply. Eventually, it will lead to cerebral ischemia, insufficient oxygen uptake, and necrotic changes in the brain [1]. The identification of neurological dysfunction is very important for the early diagnosis of cerebral infarction so that blood circulation can be improved as soon as possible. For its clinical treatment, thrombolytic therapy is the most important method, and recombinant tissue plasminogen activator (rt PA) and urokinase are widely used thrombolytic drugs in clinic [2]. The time of rt PA treatment is 3–4.5 hours, and urokinase treatment takes 6 hours. Herpich and Rincon [3] showed that the treatment was effective within 6 hours of onset, but it was life-threatening after 6 hours. Due to the long onset time of patients, thrombolysis symptoms are mainly confused with clinical symptoms. Despite the limited treatment period for cerebral infarction, the traditional equipment for predicting cerebral blood flow and cerebral thrombosis is inaccurate. Patients not following the thrombolysis therapy can result in severe bleeding [4, 5].

According to the time and degree of vascular stenosis, there may be hypoperfusion tissue at the necrotic edge around the core infarction area. If the blood supply is restored in time, it is called ischemic penumbra (IP) [6]. The concept of IP is the theoretical basis of thrombolytic therapy. The thrombolysis is to protect ischemic shadow, preserve ischemic brain tissue as much as possible, and improve the prognosis of patients. Hence, it is important for clinical treatment to quickly detect the presence or absence of ischemic shadow [7, 8]. In recent years, computed tomography perfusion (CTP) imaging is the main method to evaluate the condition of acute ischemic stroke (AIS), although magnetic resonance imaging (MRI) is more accurate in examining ischemic brain tissue [9, 10]. Cerebral blood volume (CBV), cerebral blood flow (CBF), time-to-peak (TTP), mean transit time (MTT), and other pseudocolor perfusion parameters can be used to determine the condition of ischemic brain tissue [11, 12].

CT is a new technology that combines the most advanced technologies in many fields, such as radiation, informatics, microelectronics, and computer science. In 2004, the DRL reconstruction algorithm is constructed based on the common neural network algorithm. With the pervasiveness of computer technology, artificial intelligence (AI) algorithm has been widely used in the medical field. According to some research, the AI algorithm can improve the efficiency of refinement of the CT image of the lung. The DRL algorithm is mainly based on the deep convolutional neural network (DCNN) model for low-sampling image restoration. Simply put, DCNN model is a tool for image analysis and reconstruction using backward tracking propagation algorithm including backward analysis. Because CTP image data have three-dimensional data of height, weight, and time series, a DCNN model named DRL with three-dimensional convolution layer and active layer is constructed [13]. The DRL algorithm features accurate CT reconstruction using projection data truncated along the horizontal direction of the detector. Therefore, the DRL algorithm can be used to segment ROI [14]. DRL can obtain real high-resolution sectional images and analyze images quantitatively. To reconstruct images, the imaging system uses reconstructors to receive sampled and digitized X-ray data. Moreover, it can reduce the reconstruction time and radioactivity. Although CT technology has been developed for many years and the hardware has been greatly improved, the DRL reconstruction algorithm is scarcely used to process brain images [15].

In this study, the DRL model was optimized and applied in practice. Artificial intelligence-based CT image processing can improve the effectiveness and safety of the treatment for acute cerebral infarction. CTP images were analyzed factoring in peak signal-to-noise ratio to judge noise reduction efficiency and to study the characteristics of cerebral blood flow and dark zone of cerebral blood flow in acute cerebral infarction. The study was aimed at providing the theoretical references and basis for the clinical treatment of acute cerebral infarct.

## 2. Materials and Methods

### 2.1. Research Subjects

A total of 100 patients with cerebral infarct with unknown time of onset were selected as the research subjects. All patients underwent thrombolytic therapy. According to different image processing methods, the patients were divided into the algorithm group (artificial intelligence algorithm-based image processing group) and the control group (conventional method-based image processing group). Among 50 patients in the control group, there were 24 male patients and 26 female patients, and their average age was 58.0 ± 3.22. In the algorithm group, there were 28 male patients and 22 female patients, and their average age was 56.8 ± 3.41. The differences in general data on the patients in the two groups showed no statistical meaning (*P* > 0.05). This study had been approved by Ethics Committee of hospital. The patients' family members had signed informed consent forms.

Inclusion criteria were as follows: (i) CT examination of clinical symptoms of stroke; (ii) occlusion of large vessels in anterior circulation; (iii) MRI examination showed seizure symptoms; (iv) no intracranial hemorrhage was detected in brain CT. Besides, no obvious low-density shadow corresponding to neurologic impairment was found; (v) thrombolytic therapy could be performed within 6 hours after onset. The therapy for progressive stroke could be extended to 12 hours after onset; and (vi) patients and their family members had signed and consented.

Exclusion criteria were as follows: (i) patients who did not sign informed consent; (ii) patients with brain diseases such as intracranial hemorrhage and epilepsy; (iii) patients under 18 years old; (iv) with allergy to contrast agents; (v) acute cerebral infarct, subacute bacterial endocarditis, acute pericarditis, and severe heart failure came into being in the recent 90 days; and (vi) intracranial aneurysm, arteriovenous malformation, intracranial tumor, and suspected subarachnoid hemorrhage did not occur.

### 2.2. The Algorithm Processing of CT Perfusion Imaging

The noise model in the low-dose CT projection was regarded as the Gaussian noise with the mean-variance relationship, so the mathematical expression of the noise model was expressed as follows:

(1) $$\begin{array}{c} \sigma_{i}^{2}=\frac{1}{P_{i0}}exp\left(\bar{a_{i}}\right)\left[1+\frac{1}{P_{i0}}\left(\bar{a_{i}}\right)\left(\sigma_{e}^{2}-1.25\right)\right]. \end{array}$$

In (1), *P*_*i*0_ represented the intensity of the incident ray, $\bar{a_{i}}$ represented the mean value of the chordal graph of ray penetration path *i*, *σ*_*i*_^2^ represented the variance of the chordal graph of ray penetration path *i*, and *σ*_*e*_^2^ represented the variance of the background electronic noise.

The adaptive chordal graph restoration method was used to reduce the noise and artifacts in the reconstructed CT images. In (2), the penalized weighted least squares (PWLS) were used to recover the projected data.

(2) $$\begin{array}{c} \Phi \left(p\right)={\left(y-p\right)}^{T}\sum {\left(y-p\right)}^{-1}+\beta R\left(p\right). \end{array}$$

In equation (2), *p* expressed the ideal chordal graph data to be estimated, *R*(*p*) expressed the penalty term, and *β* expressed the smoothing parameter.

According to the probability theory in the statistical analysis, the median distribution was used to predict the central tendency ratio to the mean. Then, (3) showed the calculation of *R*(*p*).

(3) $$\begin{array}{c} R\left(p\right)=\frac{1}{2}\sum_{i}{\left(p_{i}-p{\left(N_{i}\right)}_{\text{median}}\right)}^{2}. \end{array}$$

In (3), *N*_*i*_ represented the four-neighborhood pixels in the two-dimensional chordal graph gridding, and *p*(*N*_*i*_)_median_ represented the mid-value.

The modified iterative Gauss–Seidel method was adopted to optimize and solve the PWLS function.

(4) $$\begin{array}{c} p_{i}^{m+1}=\frac{1}{1+\beta \sigma_{i}^{2}}\left[y_{i}+\beta \sigma_{i}^{2}\cdot p^{m}{\left(N_{i}\right)}_{\text{median}}\right]. \end{array}$$

In equation (4), *m* expressed the number of iterations.

To solve the problem of resolution loss in the images after the reconstruction, the adaptive projection data weighting method was adopted to process image data. If the original data containing the noise image were *y*, and the image data restored by the PWLS method were *x*, the constructed adaptive weighting framework could be expressed as follows:

(5) $$\begin{array}{c} {\tilde{y}}_{i}=\omega_{i}\cdot y_{i}+\left(1-\omega_{i}\right)\cdot x_{i}, \end{array}$$

(6) $$\begin{array}{c} \omega_{i}=\left\{\begin{array}{c} 1,\sigma_{i}^{2}\leq \lambda ,\\ 0,\sigma_{i}^{2}>\lambda . \end{array}\right) \end{array}$$

In (5) and (6), ${\tilde{y}}_{i}$ represented the image data after the weighted processing, *ω*_*i*_ represented the weight coefficient, *σ*_*i*_^2^ represented the noise variance, and *λ* represented the threshold factor.

The indexes of peak signal-to-noise ratio (PSNR), root mean square error (RMSE), and universal quality index (UQI) were used for the quantitative evaluation of the effects of image reconstruction. Equations (7)–(9) showed how the above indexes were defined [15].

(7) $$\begin{array}{c} PSNR=20log_{10}\left(\frac{{\bar{f}}_{\text{max}}}{\sigma }\right), \end{array}$$

(8) $$\begin{array}{c} RMSE=\sqrt{\frac{\left[\sum_{i=1}^{n}{\left(f_{i}-{\bar{f}}_{i}\right)}^{2}\right]}{n}}, \end{array}$$

(9) $$\begin{array}{c} UQI=\frac{\left[2Cov\left(f-\bar{f}\right)\cdot 2\mu_{f}\mu_{\bar{f}}\right]}{\left[\left(\sigma_{f}^{2}+\sigma_{\bar{f}}^{2}\right)\cdot \left(\mu_{f}^{2}+\mu_{\bar{f}}^{2}\right)\right]}. \end{array}$$

In Equations (7)–(9), *f* expressed the perfusion parameter diagram after the restoration of the sequence, $\bar{f}$ expressed the reference to the perfusion parameter diagram, and *σ* expressed the standard deviation. *σ*_*f*_^2^ and $\sigma_{\bar{f}}^{2}$ expressed the contrast parameter in the region of interest and the variance of the reference parameter diagram. *μ*_*f*_^2^ and $\mu_{\bar{f}}^{2}$ expressed the contrast parameter in the region of interest and the mean value of the reference parameter diagram.

**Figure 1:**
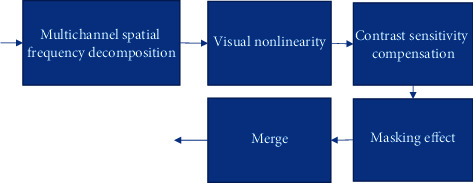
Abstract model of visual system.

The abstract model of visual system is shown in Figure 1.

### 2.3. Thrombolytic Scheme

According to *the Chinese Guidelines for the Diagnosis and Treatment of Acute ischemic Stroke 2014* [16], patients who met the inclusion and exclusion criteria accepted intravenous thrombolysis therapy within 4.5 hours of onset. *Usage.* Urokinase 0.9 mg/kg (the maximum dosage is 90 mg) was injected intravenously, and the dosage was 10% within 10 minutes. At the same time, the patient was monitored for physiological information, and abnormal physiological characteristics required symptomatic treatment. Aspirin 300 mg was taken orally 24 hours after thrombolytic therapy. Patients needed to be injected with 1 million to 1.5 million IU urokinase intravenously within 6 hours of onset.

### 2.4. CTP Examination Process

Spiral CT was employed to examine and scan the patients. Prior to the scanning, the patients needed to understand related precautions, and it should be ensured that no patients suffered from the contraindications of the examination. Before the operation, their height and weight should be recorded accurately. And trocar was inserted into anterior elbow vein. During the examination, the patients were instructed not to tilt their heads. Besides, they were told to keep body still without speaking or swallowing. In this case, good imaging effects could be obtained. The layer thickness of the scanning on head was required to be 5 mm, tube current was 100 mA, and tube voltage was 120 KV. CTP scanning was performed in dynamic reciprocating scanning mode. 40–50 mL of fluorine nonionic contrast agent was injected into anterior elbow venous double-tube high-pressure syringe at the flow rate of 4-5 mL/s. Besides, 20 mL of normal physiological saline was injected. CTP scanning was performed from the top of head to the side of foot, which lasted for 45 seconds and about 20 cycles. Tube voltage, tube current, scanning thickness, and matrix size were set to be 80 kV, 125 mA, 5 mm, and 512 × 512, respectively. The images scanned by CTP were transmitted to the perfusion analysis software at Brain Perfusion 4.0 EBW workstation by intelligent algorithm. After that, the software automatically output the images of cerebral perfusion parameters. Next, abnormal perfusion areas were divided by 3 doctors with more than 5 years of experience. The disagreement was settled by the negotiation with senior physicians.

### 2.5. Efficacy Evaluation

At one day, one week, half a month, and one month after the thrombolytic therapy, the NIHSS score was recorded, which was used as the evaluation standard of thrombolytic effect, and patients were evaluated for the brain nerve defects. One-month or three-month follow-up can reflect the long-term clinical results of thrombolytic therapy, while the NIHSS score can be used to evaluate patients' prognosis, and score ≤2 can be used as the obstacle threshold. A score of 0–2 means good prognosis, and a score of 3–4 means poor prognosis.

### 2.6. Safety Evaluation

After thrombolytic therapy combined with the NIHSS score (above 4 points), as well as CT or MRI examination, whether there is symptomatic cerebral hemorrhage is used as a safety evaluation index. In addition, the intracranial hemorrhage rates before and after thrombolytic therapy were compared. All indicators are recorded and classified by two doctors in strict accordance with the evaluation criteria to ensure the uniformity and accuracy.

### 2.7. Statistical Methods

GraphPad prism 5.0 software is used to process data expressed as mean ± standard deviation. SPSS 20.0 is used to analyze the differences among groups. *t*-Test for independent samples is performed for blood vessel diameter experiment and angiogenesis experiment. There is a statistical difference at *P* < 0.05.

## 3. Results

### 3.1. Comparison of Image Quality between Algorithm Group and Control Group

**Figure 2:**
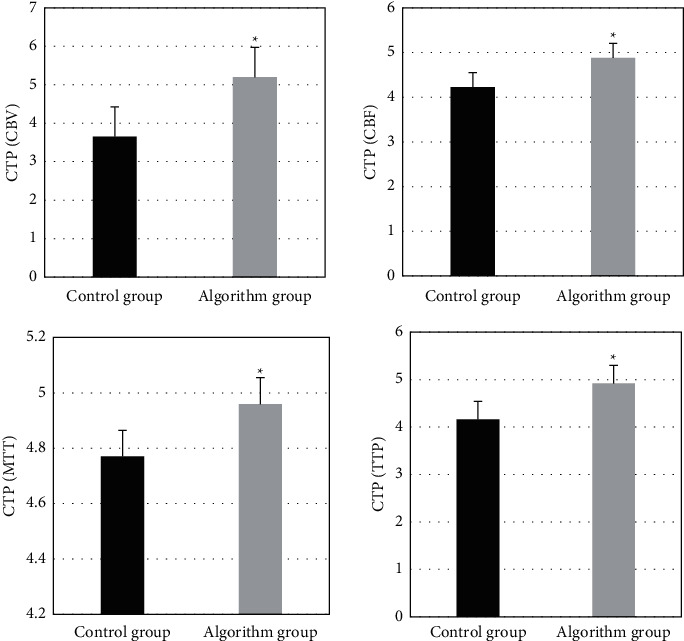
Comparison of accuracy of CBV, CBF, MTT, and TTP images between the two groups. ^*∗*^ indicates statistically significant differences (*P* < 0.05).

The pseudocolor image quality of CTP was evaluated factoring into the scores of CBV, CBF, MTT, and TTP. The image quality is divided into three grades: high (score >4), medium (score ≤4 and ≥2), and poor (score <2). As shown in Figure 2, for the image quality of the algorithm group, CBV was 5.2, CBF was 4.88, MTT was 4.96, and TTP was 4.92. The overall quality score was greater than 4, indicating high image quality. For the image quality in the control group, CBV was 3.65, CBF was 4.23, MTT was 3.77 MTT, and TTP was 4.16, with medium- and high-quality images. The overall image quality was better in the algorithm group.

**Figure 3:**
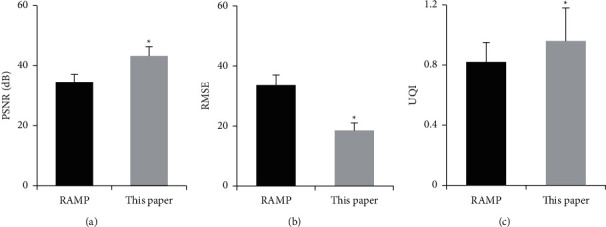
.Comparison of quantitative indexes of the images after the reconstruction. (a) comparison of PSNR; (b) comparison of RMSE; (c) comparison of UQI. ^*∗*^ indicates statistically significant differences (*P* < 0.05).

The differences in PSNR, RMSE, and UQI indexes of patients' images processed by the compressed sensing reconstruction algorithm and the proposed algorithm were quantitatively evaluated. In Figure 3, compared with the compressed sensing reconstruction algorithm, PSNR and UQI values of images reconstructed by the proposed algorithm were remarkably higher, while the RMSE values were signally lower (*P* < 0.05).

### 3.2. Comparison of the Pseudocolor CTP Images Processed in the Algorithm Group and the Control Group

**Figure 4:**
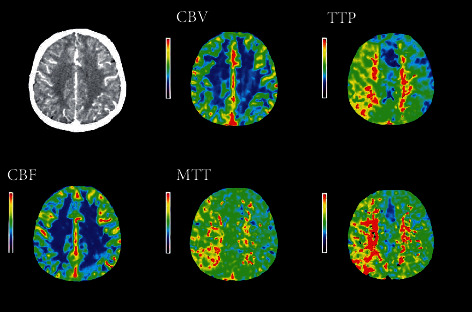
CTP pseudocolor image after processing by the algorithm.

**Figure 5:**
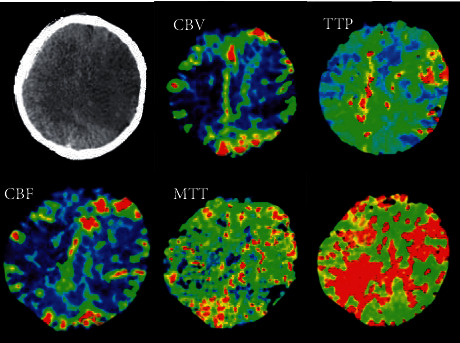
CTP pseudocolor image of control group after processing.

**Figure 6:**
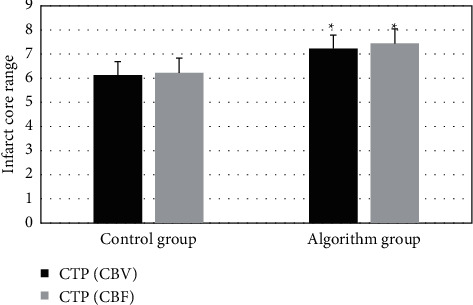
Comparison of myocardial infarction range between the two groups. ^*∗*^ indicates statistically significant differences (*P* < 0.05).

The pseudocolor CTP images of the two groups were compared for the ischemic area. The image of ischemic area and infarct core in perfusion image was very different from the normal image. As shown in Figures 4 and 5, the infarct core area in the image of the control group was slightly larger than that of the algorithm group, showing significant differences. In the algorithm group, the ischemic area and infarct core area in the original image were easier for doctors to distinguish, helpful for doctors to diagnose infarct lesions and blood flow.  Figure 6 showed the myocardial infarction foci between the two groups, and it was noted that the algorithm group was more accurate in dividing the infarction area.

### 3.3. Comparison of NIHSS Scores before and after Thrombolysis between the Two Groups

**Figure 7:**
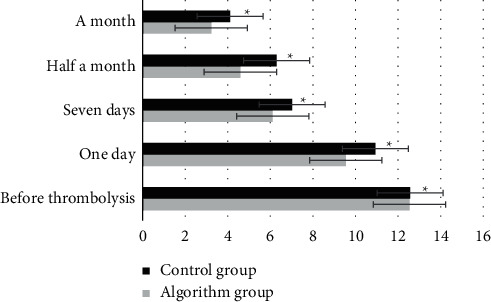
The NIHSS scores of the two groups at one day, one week, half a month, and one month after the treatment. ^*∗*^ indicates statistically significant differences (*P* < 0.05).

Figure 7 showed the NIHSS scores in the two groups. There was no significant difference between the control group and the algorithm group before thrombolysis (*P* > 0.05). On the day after thrombolytic therapy, the difference of the NIHSS scores between the algorithm group and the control group was 1.39, which was statistically significant (*P* < 0.05). One week after thrombolytic therapy, the difference of the NIHSS scores between the algorithm group and the control group was 0.91, which was statistically significant (*P* < 0.05). Half a month after thrombolytic therapy, the difference of the NIHSS scores between the algorithm group and the control group was 1.7, which was statistically significant (*P* < 0.05). One month after thrombolytic therapy, the difference of NIHSS scores between the algorithm group and the control group was 0.89, which was statistically significant (*P* < 0.05).

### 3.4. Comparison of Benign Prognosis Rate between Two Groups before and after Thrombolysis

**Figure 8:**
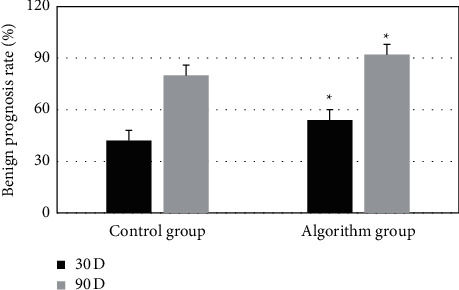
Comparison of benign prognosis rate between two groups before and after thrombolytic therapy. *∗* indicates statistically significant differences (*P* < 0.05).

As shown in Figure 8, one month after the thrombolytic treatment, the difference of benign prognosis rates between the algorithm group and the control group was 12%, with statistical differences (*P* < 0.05). After three months, the difference of benign prognosis rates between the algorithm group and the control group was 12%, with statistical difference (*P* < 0.05).

### 3.5. Comparison of Bleeding Conversion Rate between Two Groups before and after Thrombolysis

**Figure 9:**
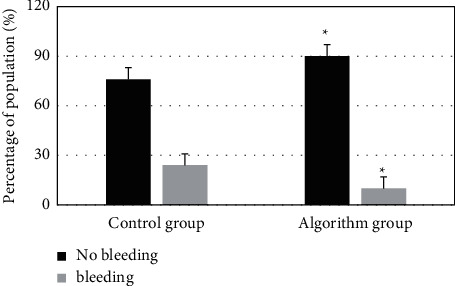
Comparison of bleeding conversion rate between two groups before and after thrombolytic therapy.

As shown in Figure 9, the symptomatic intracranial hemorrhage rate in the control group was 24% within 24 hours, and that was 10% in the algorithm group. The conversion rate of the algorithm group was lower than that in the control group, and the difference was statistically significant (*P* < 0.05).

### 3.6. Comparison of Relative Values of Parameters in Ischemic Penumbra between Two Groups

**Figure 10:**
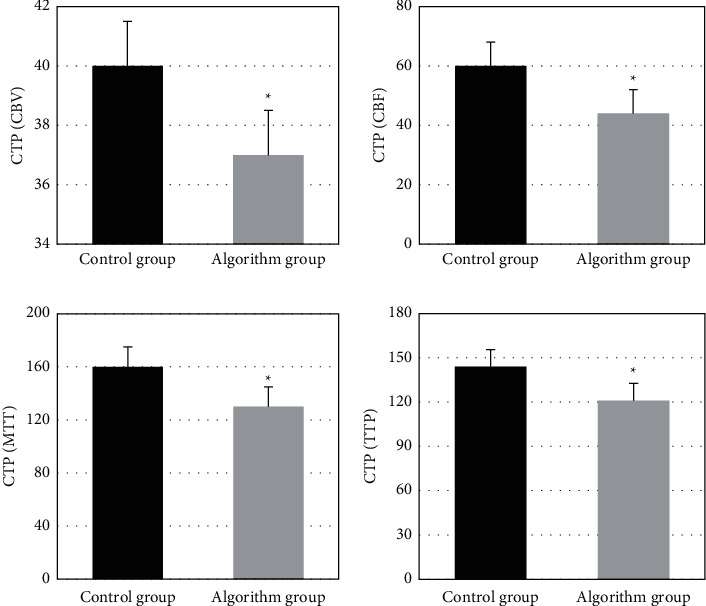
Comparison of parameters in ischemic penumbra of CBV, CBF, MTT, and TTP between two groups. ^*∗*^ indicates statistically significant differences (*P* < 0.05).

Figure 10 shows the relative values of CBV, CBF, MTT, and TTP in ischemic penumbra between the two groups. It was found that the values in the algorithm group were significantly lower than the control group, with statistical differences (*P* < 0.05).

## 4. Discussion

The incidence of acute cerebral infarction has increased in recent years, with higher mortality and disability rate, which has affected residents' health and economic level [17]. CTP is a functional imaging that shows hyperacute abnormal perfusion areas of the brain. Related studies found that the abnormality appeared 30 minutes after ischemia [18]. Deep learning-based intelligent algorithm was applied in CTP imaging, and the results showed that the image quality was apparently superior to the quality of conventional CTP images. Artificial intelligence algorithm-based CTP images demonstrated remarkable diagnostic effects. In terms of the quality of the images in the algorithm group, CBV, CBF, MTT, and TTP reached 5.2, 4.88, 4.96, and 4.92, respectively. The values suggested that the scores for image quality were high. As to the quality of the images in the control group, CBV, CBF, MTT, and TTP amounted to 3.65, 4.23, 3.77, and 4, 16, respectively. Obviously, the overall quality of images in algorithm group was relatively higher, and the data differences between the two groups revealed statistical meaning (*P* < 0.05). The results indicated that deep learning-based CTP imaging was more beneficial to the diagnosis of diseases. CTP could offer clearer images for the analysis of diseases. Ischemic penumbra relative values of CBF images in the two groups were compared. The difference of the data between the two groups was 16. Ischemic penumbra relative values of MTT images in the two groups were compared, and the difference was 30. The values between the two groups showed statistical differences (*P* < 0.05). Besides, ischemic penumbra relative values of TTP images in the two groups were compared, and the difference was 23. The values between the two groups revealed statistical differences (*P* < 0.05). The above results indicated that the 4 parameters in ischemic penumbra of algorithm group were all decreased compared with those of the control group after thrombolysis, which suggested that infarct areas were correspondingly reduced and the therapy was effective.

Zhong et al. [19] proposed an accurate CT reconstruction algorithm based on back-projection filtering, which can accurately reconstruct the cross-sectional image of the object using the minimum projection data. DRL image optimization model has fewer learning cycles, shorter processing cycles, and higher image quality after processing, which can better meet clinical needs. Then, the quality of CTP pseudocolor images was evaluated in two groups, factoring into the scores of CBV, CBF, MTT, and TTP. The image quality is divided into three grades: high (score >4), medium (score ≤4 and ≥ 2), and poor (score <2). In the algorithm group, the image quality score was greater than 4, indicating high image quality. The overall image quality in the algorithm group was better. Based on the PSNR parameter, the noise in the image can be linearly indicated. The PSNR of DRL image was relatively high. In comparison of CTP pseudocolor images of two groups, the ischemic area and infarct core in the algorithm group were quite different from that of normal image. The ischemic area and infarct core area in the algorithm group were easier to identify, assisting doctors in diagnosing infarct focus and blood flow [20, 21]. The thrombolytic effect of the algorithm group was better than that of the control group. Thrombolysis demonstrated good curative effects on the nerve function defect in the algorithm group. The symptomatic intracranial hemorrhage rate in the control group was 24%, and that in the algorithm group was 10% within 24 hour, with a difference of 14% between the two groups, showing statistically significant differences (*P* < 0.05), indicating that thrombolytic therapy under the guidance of the algorithm group was safer than that in the control group. The limitation of this study is that CTP data need to be combined with MRI data to jointly evaluate the infarct core and ischemic range. In the future research, the data will be revised twice by combining MRI scan to expand the accuracy in the diagnosis of patients with acute ischemic stroke.

## 5. Conclusion

Artificial intelligence algorithm-based CTP images showed remarkable diagnostic effects. The results of the image quality in algorithm group revealed that the scores for image quality were high and the overall quality of the images in algorithm group was relatively higher. The differences in the NIHSS scores for the two groups showed that the thrombolytic effect on the algorithm group was superior to that on the control group. After the thrombolysis of algorithm group, the therapeutic effect on neurologic impairment was significant. The symptomatic intracranial hemorrhage rate of the algorithm group within 24 hours was dramatically superior to that of the control group. The deep learning-based algorithm group-guided thrombolytic therapy showed high safety and good clinical application prospects. Besides, it possessed guiding significance to the diagnosis of CTP images of patients with cerebral infarct. In addition, the sample size is small, which will reduce the power of the study. In the follow-up, an expanded sample size is necessary to strengthen the findings of the study [22].

## Data Availability

The data used to support the findings of this study are available from the corresponding author upon request.
